# Gut Microbiota, Antipsychotics, and Metabolic Alterations in Children and Adolescents: Protocol for a Longitudinal Observational Study

**DOI:** 10.2196/77374

**Published:** 2025-12-29

**Authors:** Marcela França Dias, Ana Paula de Alcântara Freitas, Suzana Figueiredo Collares, Renata Maria Silva Santos, Yago Jean de Almeida Nogueira, Antônio Márcio de Ávila Júnior, Tamires Coelho Martins, Paulo Marcos Brasil Rocha, Marco Aurélio Romano-Silva, Débora Marques de Miranda

**Affiliations:** 1 Scientific Research Program Molecular Medicine, Universidade Federal de Minas Gerais, Belo Horizonte, Brazil; 2 Department of Mental Health, Universidade Federal de Minas Gerais, Belo Horizonte, Brazil; 3 Department of Pediatrics, Universidade Federal de Minas Gerais, Av. Alfredo Balena 190, Room 114, Belo Horizonte, 30130-100, Brazil, 55 31-3409-9753

**Keywords:** psychiatric disorders, gut microbiota-brain axis, microbiome, metabolome, second-generation antipsychotics

## Abstract

**Background:**

Over the past decade, numerous studies have emphasized the important role of gut microbiota (GM) in maintaining the body’s homeostasis. Imbalances in GM have been linked to many dysfunctions, such as metabolic and neurodevelopmental disorders. GM can be influenced by many factors, among them the use of certain medications, such as second-generation antipsychotics (SGAs), and, in turn, act upon the endocrine, immune, and nervous systems. Despite the growing interest in the microbiota-gut-brain axis, significant gaps remain in our understanding of how SGAs affect GM and the host metabolic profile.

**Objective:**

This study aims to build on the current knowledge on the impact of SGAs on clinical parameters, microbial and metabolic profiles, and behavior of children and adolescents undergoing treatment with SGAs.

**Methods:**

This is a prospective longitudinal study, in which the effects of SGAs will be assessed before and 3 to 6 months after their introduction. An integrated approach will be used, encompassing clinical data (such as weight, lipid profile, and glucose levels); microbiome and metabolome analyses; emotional, behavioral, and sleep patterns (assessed through psychiatric scales); and dietary habits.

**Results:**

This project was funded in November 2023 and will start data collection in January 2026. It is expected to be completed in 2027.

**Conclusions:**

This study is expected to provide insights into the multidimensional effects of SGAs on children and adolescents, including clinical data, GM microbial profile, metabolism, and behavior. The findings may contribute to a better understanding of treatment impacts and provide information on more personalized therapeutic strategies.

## Introduction

Communities of microbes, collectively known as microbiota, have evolved in close association with their hosts and colonize various regions of the human body, with the highest concentrations in the gastrointestinal tract [1]. Growing evidence supports the relevance of GM to the host’s health. It is involved in a wide range of physiological processes, such as nutrient metabolism, gastrointestinal motility, immune regulation, and even neurological functioning [1,2]. On the other hand, GM composition is influenced by numerous host-related factors, including age, lifestyle, and pharmacological intervention, such as antipsychotic medications [3], which are the focus of this study.

In early life, GM is particularly susceptible to environmental factors, with long-lasting effects on immune, metabolic, and neuroendocrine pathways [4]. Perturbations during key developmental stages could predispose children to different conditions through immune dysregulation and altered neurodevelopmental signaling. These connections are yet to be understood, and they highlight the importance of studies focused on pediatric populations.

The use of antipsychotics, especially second-generation antipsychotics (SGAs), has extended far beyond their primary indications and is increasing among children aged as early as 3 years [5]. SGAs are prescribed for off-label conditions including anxiety, autism, and attention-deficit/hyperactivity disorders [5,6]. Despite its therapeutic potential, the use of SGAs in youth is associated with important side effects, which include weight gain, metabolic disturbances, heart disease, and sedation, among others [7-9].

Children and adolescents appear to be particularly susceptible to antipsychotic-induced impacts [10]. These side effects can emerge rapidly and be widespread, with studies reporting a notable increase in weight within the first 15 weeks of treatment [11] and clinically relevant weight gain (≥7%) associated with almost all antipsychotic medications investigated [12]. Notably, agents considered neutral in adults may lead to weight gain in younger populations, as discussed elsewhere [10,13].

Given the complex nature of the effects of SGAs, comprehensive monitoring, which addresses the interaction between SGAs and clinical, microbial, metabolic, and behavioral data, is important for developing safer and more personalized treatment strategies. In accordance, the primary purposes of this study are to evaluate changes in key clinical parameters, GM composition, and metabolic profile in children and adolescents before and after treatment (3 to 6 months) with SGAs. The secondary goals are to assess behavioral, emotional, sleep, and dietary parameters and to explore their potential associations with microbial, metabolic, and clinical changes. For that, the following specific objectives were defined:

- Evaluate changes in weight, BMI, abdominal circumference, glucose levels, and lipid profiles at the 2 time points
- Compare GM and metabolic profiles before and after SGA therapy
- Evaluate behavior, emotional regulation, and sleep quality using validated psychometric tools (Emotional Regulation Checklist, Adolescent Behavior Inventory, and Pittsburgh Sleep Quality Index), previously adapted to the population under study
- Investigate changes in dietary patterns before and after treatment and their associations with the evaluated parameters
- Explore, through statistical modeling techniques, potential connections between the studied factors and the introduction of SGAs and other variables that might influence the results, such as drug and age.

In this way, this study aims to address a current gap in the literature, as no previous research, to the best of our knowledge, has yet integrated clinical parameters with metabolome and microbiome data in the context of SGA therapy. The results could help identify associations that may, in the future, contribute to elucidating the mechanisms through which GM could be associated with the side effects of these medications.

## Methods

### Study Design

This prospective observational study will evaluate the effects of SGAs in children and adolescents at 2 time points: before (baseline) and 3 to 6 months after treatment initiation. No intervention will be applied, and all data will be collected as part of participants’ routine clinical care, thereby allowing the monitoring of temporal changes in metabolic, microbial, behavioral, and clinical parameters associated with the use of SGAs. This study adheres to the STROBE (Strengthening the Reporting of Observational Studies in Epidemiology) guidelines [14], which provide a structured framework for transparent and comprehensive reporting of observational research.

### Study Population

Participants will be recruited from the General Child Psychiatry, Autistic Spectrum Disorder, and Attention-Deficit/Hyperactivity Disorder outpatient clinics of the Hospital das Clínicas of the Universidade Federal de Minas Gerais. Eligible participants will include children and adolescents, aged 6 to 17 years, who have a clinical indication for antipsychotic treatment but have not yet started it.

### Sample Size

In this observational study, participant recruitment will occur progressively, depending on the availability of eligible individuals initiating SGA therapy in the clinical setting. This design aligns with routine clinical practice; however, it may influence the number of participants recruited over time. The sample size estimation was calculated with 1-sample *t* test [15,16], using differences in body weight before and after treatment as the primary outcome, given its robust and early response to SGAs.

Statistical power and significance level were set at 0.80 and 0.05, respectively, which are commonly accepted values [15,16]. An effect size of 0.58 was estimated based on unpublished data from a previous study conducted by our research group. Given the limited sample size of the previous dataset and the proximity of this estimate to the conventional threshold for a medium effect size, a more conservative Cohen d_z_ [16] of 0.5 was adopted for the calculation. Based on this assumption, a minimum of 34 participants is required.

The previous data also indicated a dropout rate of approximately 23% (7 of the 30 recruited participants did not bring any samples, did not return for the second sampling at the due time, or interrupted SGA treatment). Thus, considering this information, the final number of participants required for the study is 45 [15]. As GM and metabolome data become available, this estimate may be refined.

### Data Collection

Recruitment will take place during patients’ routine medical visits. If SGA treatment is indicated, the research will be explained to parents and patients, and upon both parental consent and child’s assent, blood and feces samples will be collected before and approximately 3 to 6 months after the therapy starts. During the initial visit, after recruitment, patients’ height, weight, and abdominal and hip circumferences will be measured. Dietary patterns questionnaires, kindly provided by the South American Youth/Child Cardiovascular and Environmental Study group [17], will be completed by the parents or the child and returned to the researcher.

To minimize discomfort, blood samples will be collected alongside routine laboratory tests (fasting glucose, glycated hemoglobin, total cholesterol and fractions, and triglycerides), scheduled for the follow-up appointment, with no need for additional venous access. For metabolomic analysis, plasma will be extracted from blood samples conditioned in 4-ethylenediaminetetraacetic acid tubes by centrifugation at 3000 g for 15 minutes at 4°C and divided into as many as possible 300 µL aliquots. Metabolomics will be conducted through an academic partnership using liquid chromatography coupled with mass spectrometry [18].

Regarding fecal samples, a collection kit (Coloff) will be provided during the same visit in which the laboratory tests will be requested, along with detailed instructions for use and storage. Fecal collection may be carried out at home, preferably on the same day or the day before the blood tests. Participants will be instructed to maintain the material under refrigeration (2°C to 8°C) until delivery to the research staff, which will then be aliquoted into 0.25 µg and maintained at −80°C until further processing. For microbiome analysis, total DNA will be extracted from fecal samples using the QIAamp PowerFecal Pro DNA Kit (Qiagen), according to the manufacturer’s instructions. The concentration and quality of the extracted DNA will be assessed with the NanoDrop 2000 spectrophotometer (Thermo Fisher Scientific). Shotgun metagenomics will be performed on the Illumina platform by a sequencing service provider to be defined (paired-end 150 bp), with at least 6 GB of data per sample.

Given the limited evidence supporting behavioral improvements solely attributable to antipsychotics in youth and the significant risk of adverse effects, behavioral changes will also be investigated. This approach will be carried out using validated and standardized instruments that assess the emotional and behavioral status and sleep patterns of each participant, as reported by caregivers and patients. Key assessment tools include the following:

- Child and Adolescent Behavior Inventory: a broad-spectrum tool for evaluating behavioral and emotional symptoms in children and adolescents aged 6 to 18 years, which has been validated for the Brazilian population [19,20]. It covers multiple domains, including anxiety, depression, attention problems, and conduct disorders.
- Emotion Regulation Checklist: this tool assesses emotional functioning, focusing on regulation and lability or negativity; it has also been validated for the Brazilian context in children aged 3 to 12 years [21,22].
- Pittsburgh Sleep Quality Index: This questionnaire assesses various dimensions of sleep quality over a 1-month period [23]. It has been validated for use in Brazilian children and adolescents [24] and is particularly useful for identifying sleep disturbances.

### Data Analysis

#### Microbiome and Metabolome

Metagenomic data analysis involves several steps using different bioinformatic tools. Initially, quality control will be performed to remove low-quality sequences and adapter contamination. FastQC [25] will be used for quality assessment and Trimmomatic [26] for trimming. Host DNA will be removed by alignment to the host genome using Bowtie2 [27]. Filtered reads will then be assembled into contigs with metaSPAdes [28], followed by gene prediction using Prodigal [29]. Taxonomic profiling will be conducted using Kraken2 [30]. Functional profiling will be assessed with DIAMOND [31] in combination with UniProtKB [32], followed by mapping to the KEGG (Kyoto Encyclopedia of Genes and Genomes) Orthology system [33,34].

Statistical analysis and data visualization will be conducted to explore community structure, diversity, and differential features across samples, using *R* packages including Phyloseq [35], Vegan [36], and DESeq2 [37]. Differentially abundant and clinically relevant microorganisms identified before and after the beginning of antipsychotic therapy will be quantified using quantitative PCR to determine their absolute concentrations and improve experimental reproducibility. Specific primers will be selected from the literature, and standard curves will be constructed for each target.

Metabolomic data will be processed using the R package XCMS [38] for peak detection, alignment, and normalization. Subsequent univariate and multivariate statistical analyses will be conducted in MetaboAnalyst [39], and significant metabolites will be annotated against curated databases, including KEGG [33], Lipid Maps [40], and the Human Metabolome Database [41]. This approach will enable comparisons between time points and the identification of metabolic patterns associated with the treatment.

#### Psychiatric Scales and Nutritional Data

The psychiatric scales were selected based on their specific indications, with each scale’s scoring system used to assess the presence and severity of symptoms. The results will be analyzed together with other variables, such as clinical, microbiological, and metabolic data, to provide a comprehensive understanding of the impact of SGAs on patients’ behavior, sleep pattern, and emotional conditions.

The Dietary Determinants Questionnaire (DDQ) and the Food Frequency Questionnaire (FFQ) [17] will be used to investigate dietary habits, and the resultant information will be included in the analysis if participants present alterations in any of the other variables assessed by the study (clinical, microbiological, metabolic, or psychiatric). The DDQ will be analyzed qualitatively by thematic sections (the use of dedicated software is not planned at this stage). Items directly relevant to the study aims will be identified and dichotomously coded, for example, “consumes a given food” versus “does not consume,” and “perceives being overweight” versus “perceives being underweight,” to characterize parental practices, child/adolescent eating autonomy, and overall dietary behavior.

The FFQ will be analyzed qualitatively and categorically, based on the frequency of consumption of each food item (eg, never or up to once a week, 2 to 4 times a week, or up to once a day). This categorization will allow the identification of dietary patterns associated with the participants’ nutritional profiles, with an emphasis on relevant food groups (including ultraprocessed foods and sugar-sweetened beverages, among others). In addition, an integrated individual-level analysis will be conducted by combining FFQ and DDQ data with the clinical outcomes observed during the study.

#### Statistics

Clinical outcomes (body weight, BMI, waist and hip circumference, glucose, and lipid profiles) will be analyzed using regression-based approaches appropriate to the data distribution, with change in body weight as the primary outcome. Models will be adjusted for age, diagnosis, sampling interval, SGA type, and dose (chlorpromazine equivalents).

To integrate datasets, correlation analysis will be used to assess associations between changes in GM (diversity measures and differential abundance of clinically relevant bacterial taxa), metabolic profile, and clinical outcomes. When sample size allows, analyses will be stratified by SGA and age group. A significance level of .05 will be used, and CIs for the outcomes of interest will be provided.

### Ethical Considerations

The study protocol has been submitted and approved by the National Research Ethics Commission, Brazil (CAAE 79026224.4.0000.5149). The ethical aspects of this study have been carefully considered to ensure the protection and well-being of all participants. Potential benefits include improved understanding of the metabolic and behavioral effects of the use of SGAs, which may contribute to more personalized treatment strategies. Minimal physical risks are associated with blood collection, and potential psychological or social discomfort may arise from fecal sampling. There is no planned compensation, since this is an observational study without any intervention; however, a dedicated team will be available to provide assistance whenever necessary.

All participants and their legal guardians will receive comprehensive information about the study, including its objectives, procedures, potential risks, compensation, and benefits. Informed consent will be obtained from parents or legal guardians, along with assent from the children, before engaging in any activity related to the project. The informed consent form clearly states the voluntary nature of participation, the right to withdraw from the study at any time without penalty, and the measures to ensure confidentiality. In this regard, personal and clinical data will be anonymized and securely stored, with access restricted to authorized research personnel only, in compliance with data protection regulations.

## Results

The project was funded in November 2023 and will be conducted in accordance with the approved protocol. Data collection is projected to begin in January 2026 and run until June 2027. Results are expected to be available for publication in 2027.

Preliminary, unpublished data from a previous study conducted by our research group enabled the evaluation of participant adherence and identification of key logistical and procedural challenges. In that cohort, 30 participants were recruited; 11 provided fecal samples; 7 discontinued their participation. An approximate 23% dropout rate was observed among initially consenting, eligible individuals, mainly associated with fecal samples requirements.

In the previous study, participants were homogeneous in age (mean 8.7, SD 4.1) and gender (all male). Clinical profiles included 20 children with autism spectrum disorder, 8 with oppositional defiant disorder, and 2 with comorbid attention-deficit/hyperactivity disorder and oppositional defiant disorder. Similar proportions in diagnostic categories and gender distribution are expected in the present project.

## Discussion

SGAs are an important therapeutic option in child and adolescent psychiatry, helping to stabilize disorders such as schizophrenia, bipolar disorder, and psychotic depression. They are also used to manage other conditions, including autism, tics, and aggressive behavior in children [13]. However, antipsychotic-induced weight gain and related metabolic disturbances are frequent and clinically relevant adverse effects, which result from multiple interacting mechanisms and contribute to reduced life expectancy [10,13,42].

As discussed previously [10,13], SGAs can interfere with appetite and metabolic regulation, resulting in energy imbalances. These effects occur through different mechanisms, such as alterations in hormone levels (eg, leptin, ghrelin, adiponectin, and insulin) and neurotransmitter pathways, including dopamine D2 receptor antagonism, a common feature of antipsychotics that is associated with eating behavior [10]. Alterations in GM have also been implicated in SGAs adverse effects, as these medications can modify gut microbial composition [3], and promote, for example, inflammatory cytokine release, which could contribute to metabolic dysregulation through bidirectional gut-brain interactions [10]. Metagenomics and metabolomics, addressed by this study, may provide valuable insights into the potential associations between alterations in GM and plasma metabolites, contributing to the understanding of the biological pathways involved in SGA-related metabolic impacts.

Different strategies can be used to mitigate the undesirable effects of antipsychotics, including the use of adjunctive medications such as metformin, which may help prevent or reduce metabolic complications, including weight gain [43]. The use of metformin, though widespread, also presents challenges. Gastrointestinal side effects such as nausea, diarrhea, and abdominal discomfort are common, particularly at the beginning of treatment. Therefore, adherence can be compromised, especially among pediatric or psychiatric populations. In this context, studies on the interaction between SGAs, GM, and dietary patterns are important to inform evidence-based nutritional approaches and reduce excessive pharmacological intervention.

These considerations have led to growing interest in understanding how antipsychotic medications influence GM. For example, a study similar to the present one identified taxonomic and functional alterations on GM of overweight children in treatment with SGAs [44]. However, since dietary patterns and microbiota profiles vary substantially across populations, achieving clinically meaningful understanding of the impacts of SGAs in GM and successful microbiota-based interventions require longitudinal investigations in diverse treatment contexts.

This study presents relevant strengths, particularly its comprehensive approach, which addresses various domains of participants’ functioning that could be impacted by SGA treatment. The use of validated psychometric tools and standardized protocols for sample collection increases methodological rigor. Additionally, within-participant comparisons reduce the risk of bias related to interindividual variability. However, the study also presents considerable limitations, particularly related to sample size, constrained by the availability of eligible patients, variations in the interval between samplings, and expected dropout rates, which highlights the need for strategies that increase engagement.

An additional and important limitation is the predominance of male participants, reflecting the clinical population most frequently referred for antipsychotic treatment in the study setting. This gender imbalance restricts the generalizability of the findings, although efforts will be made to include female participants whenever possible. The assessment at only 2 time points can also be considered a limitation, as it restricts the evaluation of long-term trajectories of microbiome, metabolome, and clinical outcomes. Although a third follow-up visit would strengthen the study, feasibility constraints prevented its inclusion in this protocol. Furthermore, the observational design does not allow causal inferences, and potential confounders such as comorbidities, concurrent medications, and environmental factors may influence the outcomes despite attempts at standardization.

Therefore, although this study may bring relevant information by integrating different aspects that might be affected by SGA medications on a pediatric population, there are considerable feasibility challenges that must be acknowledged. Variations in follow-up intervals and difficulties related to participant adherence and sample collection may affect data completeness and introduce heterogeneity. The predicted sample size also limits statistical power, restricting the scope of multilevel analyses. However, this limitation is expected to lessen as a larger number of participants will allow for more precise effect size estimation. As a final point, the findings should be regarded as exploratory and hypothesis-generating, primarily aimed at identifying trends and contributing to future studies with larger cohorts.

## Abbreviations

DDQ: Dietary Determinants Questionnaire
FFQ: Food Frequency Questionnaire
GM: gut microbiota
KEGG: Kyoto Encyclopedia of Genes and Genomes
SGA: second-generation antipsychotic
STROBE: Strengthening the Reporting of Observational Studies in Epidemiology
